# Energy-Aware IoT-Based Method for a Hybrid On-Wrist Fall Detection System Using a Supervised Dictionary Learning Technique

**DOI:** 10.3390/s23073567

**Published:** 2023-03-29

**Authors:** Farah Othmen, Mouna Baklouti, André Eugenio Lazzaretti, Monia Hamdi

**Affiliations:** 1 Tunisia Polytechnic School, University of Carthage, La Marsa, Tunis 2078, Tunisia; 2 CES Lab, University of Sfax, Sfax 3029, Tunisia; mouna.baklouti@enis.tn; 3 Graduate Program in Electrical and Computer Engineering, Federal University of Technology (UTFPR), Curitiba 80230-901, Paraná, Brazil; lazzaretti@utfpr.edu.br; 4 Department of Information Technology, College of Computer and Information Sciences, Princess Nourah bint Abdulrahman University, P.O. Box 84428, Riyadh 11671, Saudi Arabia; mshamdi@pnu.edu.sa

**Keywords:** fall detection, wrist-based wearable device, IoT, Supervised Dictionary Learning, energy efficient, elderly health care

## Abstract

In recent decades, falls have posed multiple critical health issues, especially for the older population, with their emerging growth. Recent research has shown that a wrist-based fall detection system offers an accessory-like comfortable solution for Internet of Things (IoT)-based monitoring. Nevertheless, an autonomous device for anywhere-anytime may present an energy consumption concern. Hence, this paper proposes a novel energy-aware IoT-based architecture for Message Queuing Telemetry Transport (MQTT)-based gateway-less monitoring for wearable fall detection. Accordingly, a hybrid double prediction technique based on Supervised Dictionary Learning was implemented to reinforce the detection efficiency of our previous works. A controlled dataset was collected for training (offline), while a real set of measurements of the proposed system was used for validation (online). It achieved a noteworthy offline and online detection performance of 99.8% and 91%, respectively, overpassing most of the related works using only an accelerometer. In the worst case, the system showed a battery consumption optimization by a minimum of 27.32 working hours, significantly higher than other research prototypes. The approach presented here proves to be promising for real applications, which require a reliable and long-term anywhere-anytime solution.

## 1. Introduction

For millennia, humans have always sought to push the limits of life further. Indeed, life expectancy is increasing considerably. Combined with the marked falls in fertility occurring in almost every country, an amplification of the aging population is confronted by the world. Statistics provided by the United Nations [1] show that, by 2050, the global population of over 65 years is expected to reach nearly 1.5 billion people, compared to 727 million in 2020. The globally accelerating growth in the number of older persons leads to an increased population fragility and the old-age dependency ratio. The unintentional fall is one of the most severe health problems faced by this older age group, which is prominent among the external causes of morbidity or mortality as highlighted in the eleventh version of the International Classification of Disease (ICD-11) [2]. It is a major cause not only of fatal injuries that may induce death but also of dramatic psychological consequences that create a crucial hindrance to one’s living independence. Overall, significant injuries occur in 4–15% of falls, and 23–40% of injury-related deaths in older adults are due to falls, making it the second cause of death from injury globally and the fifth leading health condition associated with disability in the population aged 60 years and older [3].

To reduce the weight of the associated psychological, physical, and economic consequences caused by long lying periods after a fall, it is essential to report the occurrence of an inevitable fall as soon as possible. Therefore, the need for an assisting device for fall detection is increasing in the healthcare sector with the rapid growth of the elderly population in the world to maintain a good quality of life with a notably reduced cost of treatment. Creating an interesting and promising research area for decades, a tremendous amount of solutions were proposed to meet these requirements [4,5]. Typically, automated fall monitoring systems detect falls, trigger notification alarms, and send messages calling for the timely help. A fall detection solution has rapidly evolved as it has been explored in research and industry mainly through three forms of studies: ambient-, camera-, and wearable-based.

Over the recent years, wearable inertial sensors devices have seen a keen interest from the academic and industrial communities, especially with the acclimation of smart wearable devices and sensors in daily life, including smartphones, smartwatches (wristbands), and glasses, as well as the advent of new sensors combined with the IoT (Internet of Things) paradigm. They have mainly been stimulated not only by the need to assure privacy protection and comfort for daily usage but also by the reduced cost of multimedia content production and their anywhere-anytime availability [5]. As an example, the work presented in [6] proposed a threshold-based fall detection approach based on smartphone accelerometer data. This approach distinguishes fall events from daily activities, supporting four directions (forward, backward, left lateral, and right lateral). The proposed system was evaluated on only four young people, which can affect the accuracy of the obtained results. Moreover, the fall detection approach is based on the data acquired from the built-in accelerometers of a smartphone. Through analyzing the acceleration features, effective threshold values have been identified. Such a manner could pose problems and challenges in determining reliable threshold values when changing from one smartphone to another. It has been proved that the best on-body placement for an inertial sensors unit is near its center of mass, precisely the waist, which is aimed at characterizing human mobility robustly during a fall [7]. Therefore, most wearables for fall detection were designed to be attached to the waist, thigh, or chest [8].

### 1.1. Problem Statement and Assumption

Commonly, state-of-the-art methods for wearable fall detectors are either analytical-based or machine learning-based, for which the latter received superior interest in recent years [4], as a result of its higher efficiency achievements. In [9], authors studied the performances of some machine learning algorithms to distinguish between falls and daily living activities using extracted features from a wearable wireless node composed of a gyroscope and triaxial accelerometer sensors. This work is based on online classifications using a fog computing environment. An evaluation of classification performances has also been conducted targeting both learning and run-time phases while comparing different classifiers in detection and fall pattern recognition. To ensure online computing of the optimized classifier while guaranteeing the best testing time in these experiments, the k-Nearest Neighbors (kNN) classifier was selected, providing an accuracy of 90.28%.

Furthermore, the design of an optimal combination between the data features and the classifiers to enhance system reliability has been extensively researched in most related works [10,11]. Nevertheless, the classifying performance can be largely troubled, as hand-crafted features may be very specific to the used sensor, on-body placement, or the learned dataset [12,13]. Notwithstanding this, deep learning (DL) techniques are being employed to overcome the machine learning issue. However, most DL-based systems demand a higher amount of data and do not propose a real-world prototype for the entire fall detection and rescue systems, as power consumption and real-time analysis are not normally reported in those cases [14]. Other systems have used the Generative Adversarial Network (GAN) to detect falls. In fact, Nho et al. [15] proposed a GAN-based fall detection method using a heart rate sensor and an accelerometer. It is based also on a User Initial (UI) heart rate information features to enhance the performance of the system. This study has just proposed an estimation of using the UI-GAN approach with the smartwatch, and no real experimental prototype has been evaluated.

### 1.2. Contributions

This paper is an extension of previous works [16,17]. In those works, we first introduced a Supervised Dictionary Learning approach for wrist-based fall detection [16]. The Sparse Representation-based Classifier (SRC) algorithms obtained an impressive overall performance reaching 99.8% accuracy based on an optimized solution using only a triaxial accelerometer. Second, we explored its robustness in facing overlapped and noisy data compared with shallow machine learning models (ML) [17]. All the previous work was carried out in an offline condition. No real-life prototype assessment was approved. In this paper, we propose a hybrid double prediction technique based on Supervised Dictionary Learning to reinforce the detection efficiency and assure the reliability of the wearable device. In addition, a larger scope of the proposed study is conducted by evaluating the system’s reliability and performance in real life. To this end, an end-to-end fall detection prototype has been implemented and tested.

This study will focus on three main modules: (i) a gateway-less IoT-based architecture for wearable fall detection system will be proposed for anywhere-anytime accessibility and surveillance from caregivers; (ii) an energy-aware intelligent interruption-triggering system, able to manage the energy consumption of the wearable device; and (iii) a hybrid Supervised Dictionary Learning (SDL) post-fall detection is proposed to enhance system efficiency.

Unifying these three modules into our formal theoretical study provides a more efficient and realistic fall detector. This work aims to implement an energy-aware IoT-based on-wrist fall detection system for comfortable, efficient continuous anywhere-anytime monitoring using only an accelerometer to assist the elderly in everyday life.

This paper is structured as follows: Section 2 reviews fall detection studies focusing on the type of sensors used. It also reviews existing works dealing with IoT-based systems for wearable fall detection, particularly wrist-based systems. Section 3 details our proposed IoT-based framework for wrist-based fall detection. Section 4 introduces the implementation details and provides a discussion and evaluation of the proposed system, mainly in terms of power consumption. Section 5 concludes this work with a brief outlook on future work.

## 2. Background Scenic Review

### 2.1. Analysis of Fall Detection Studies

A fall detection system is mainly composed of three fundamental stages: (i) sensing, (ii) analysis stage, and (iii) notification. The sensing stage is when the system acquires data from one or multiple sensors, such as cameras or accelerometers, to detect falls. The analysis stage is where the data should be processed and analyzed. Indeed, the most significant features are extracted from the sensors’ acquired data to identify the occurrence of falls from the other activities of daily living (ADLs) through a prediction algorithm. Once a fall event is detected, a notification must be delivered to those concerned, i.e., family members or caregivers who must be informed by e-mail, Short Message Service (SMS), or specific application notification. Most of the review papers [5,18] categorize fall detection methods depending on the type of sensors utilized in the system and their placement into three general approaches: wearable-based, vision-based, and ambient-based.

Commonly, each of these methods has its advantages and disadvantages. However, until now, they have yet to show full-scale satisfaction with its accuracy. Below, we briefly review these previously cited sensor-based approaches.

#### 2.1.1. Vision-Based Sensors

Falls can be detected using computer vision advances and image processing techniques in which camera sensors are the main actors in monitoring the human body positions, shapes, and inactivity periods by recording his daily behavior [19]. The main advantage of these vision sensors is their robustness by accurately differentiating the occurrence of falls and other ADLs. They can monitor more than one subject with less intrusion if used in public areas where multiple people need to be supervised. On the other hand, fall detection systems based on camera sensors present numerous disadvantages, like privacy evasiveness, limited area coverage, light conditions, and high material cost. Indeed, privacy and confidentiality are critical concerns since the monitored person will be watched in most of his daily activities. In addition, they cannot cover all indoor angles as the materials’ installation and maintenance are expensive.

#### 2.1.2. Ambient-Based Sensors

Ambient-based devices aim to detect a fall event by collecting and inspecting the monitored person’s movements based on his surrounding environment’s changes [19]. Various suitable sensors are placed all around the encircling indoor environment, such as the subject’s home. By tacking the floor vibrations and pressure changes combined with radio-frequency and even acoustic sensors [5], body motion diversity can be recognized passively without interfering with privacy. It is unnoticeable by the targeted person compared with the vision-based method. However, these sensors are sensitive to noises as they can be easily affected by background noises and interference, resulting in numerous false alarms. Additionally, these devices can only cover and monitor a limited area indoors as some blind spots may exist.

#### 2.1.3. Wearable Based Sensors

Wearable-based fall detection systems illustrate all on-body attached garment devices that usually embed inertial measurement units (IMUs) to inspect the body’s motions, positions, and rotation movements. Commonly, inertial sensors such as accelerometers, gyroscopes, and magnetometers are the most used for fall detection. Wearable-based fall detection systems mainly present an ideal solution for everyday monitoring (indoor and outdoor) with an acceptable price compared with vision devices. Wearable sensors can be privacy intrusive, as they must be attached to the subject’s body, especially when attached to uncomfortable body parts (chest or thigh). However, nowadays, advances in wearable technologies make it possible to embed sensors in accessories like pendants, bands, and glasses to make them more comfortable. Furthermore, modern smartphones and smartwatches are well equipped with useful sensors for a fall detection system, making them more flexible and easier to set up. However, the major disadvantage of wearable devices is power dependency as they are battery-powered, which means they must be recharged once in a while, presenting a challenging task in optimizing consumption.

In this work, we assume that wearable fall detection is the most suitable for anywhere-anytime monitoring, keeping pace with advances nowadays and users’ familiarization with wearable devices, especially when placed in accessories-like places.

### 2.2. Related Works

Different researchers have proposed energy reduction techniques to lower energy consumption either in IoT-based systems [20,21], or in Wireless Sensor Networks (WSN) [22,23]. In [22], the authors proposed a task-based model for minimizing energy consumption in WSNs. This approach analyzes possible sensor operations and generates an energy management model for the network. The proposed framework has been tested to simulate a simple WSN architecture. Further work needs to be performed to test the framework on a real case study and to consider the heterogeneity of the sensors in the network. Ref. [24] surveyed various energy-efficient techniques in the Internet of Wearable Things (IoWT). Ref. [25] also surveyed recent energy management techniques in IoT networks. It is clear that there are still many challenges to face while considering the increasing power requirement of IoT devices. The related works presented in this section will mainly focus on IoT-based systems for wearable fall detection in general, and wrist-based in particular. Notably, we have addressed the energy consumption and reliability factor as the main topics for compared related research.

Mauldin et al. [26] have developed an Android application called “SmartFall” using an accelerometer based on a smartwatch. The latter collects the data and sends it via Bluetooth Low Energy (BLE) to a smartphone that will focus on the computation process to detect falls. The smartphone application will connect to a web service to provide an updated deep learning-based fall detection model using the collected data. The authors have picked a 31.25 Hz sampling rate to reduce the computational cost. The system has shown an offline accuracy of 0.99 and an online accuracy of 0.7 using the proposed deep learning algorithm.

Ajerla et al. [27] implemented a real-time patient framework for fall detection using different body positions, including the wrist. The system interacts from an IoT device via BLE to an edge-based analytical engine using the Long Short-Term Memory (LSTM) model. The latter has proven a significant online efficiency of 95.8% using combined waist and wrist positions. Regardless, the authors did not mention the energy consumption of the proposed framework.

An energy-efficient IoT-based wearable fall detection has been proposed by Gia et al. [28]. The literature implements a lightweight, tiny, and energy-efficient wearable device as several methods are available to explore distinct aspects (i.e., transmission protocol, communication bus interface, transmission rate, and sampling rate), operating situations, and their impact on energy computation. The authors gave various hardware suggestions to embed an optimum wearable device to an IoT-based fall detection system with a higher quality of service and lower energy consumption.

In Liu et al. work [29], an energy-efficient fall detection method was proposed. The system is fixed on the waist at the front side of a vest, and an energy-efficient technique was established in the sensing module to sense and distinguish human activities from inactivity. An interruption-based technique is presented for transmitting the data to a server via the Zigbee-based gateway. A deep neural network for fall detection model was implemented on the server to detect accurate falls.

Saadeh et al. [30] introduced a single wearable sensor (using only an accelerometer) for IoT-based fall prediction. The approach presented double parallel methods, i.e., a threshold fast prediction method and a nonlinear Support Vector Machine (SVM) prediction technique. The fall detection wearable sensor is strapped to the upper thigh and transports data via BLE to predict falls. The reported fall detection offline accuracy attained 98.6%. The proposed approach has not considered the system’s energy consumption and did not present online tests.

In all of the previously described literature, most of the proposed IoT architecture is based on a low-energy end device located on the waist, a gateway-dependent transmission protocol (BLE and Zigbee), and a server (local- or internet-based). The main limitation of that architecture is that the system is principally dependent on the mediator, i.e., a smartphone or gateway to a central server.

In our work, we assume that the elderly must be supervised indoors and outdoors and are susceptible to forgetting their smartphone if the system is gateway-dependent. Indeed, a comfortable and energy-efficient autonomous architecture is needed to ensure anywhere-anytime monitoring. Therefore, we have the following original aspects that were not included in the literature altogether: (i) Mono-sensor (accelerometer only) wearable device; (ii) Gateway independent for indoor and outdoor use; (iii) Energy-aware WiFi-based system; (iv) Supervised dictionary-based learning for an improved noise-robust system; and (v) Hybrid fall and post-fall detection.

## 3. Proposed IoT-Based Framework for Wrist-Based Fall Detection

**Figure 1:**
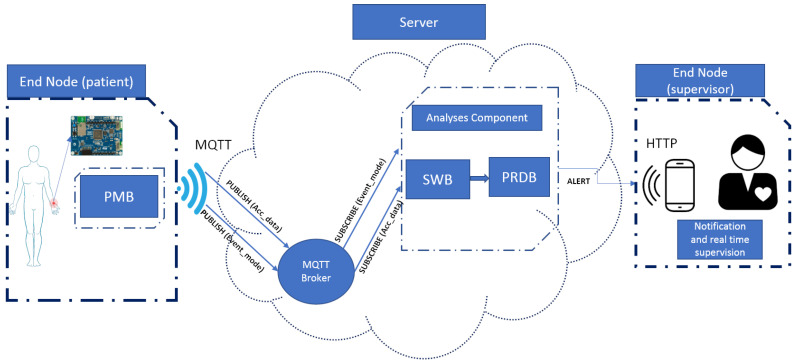
On-wrist fall detection general architecture.

This section presents a detailed demonstration of our proposed IoT-based framework for on-wrist fall detection (OWFD). The proposed framework is subdivided into two central stages: the end node (patient and supervisor) and a server. Figure 1 represents the proposed OWFD architecture, and its main block components are explained as follows.

### 3.1. Power Management Block (PMB)

**Figure 2:**
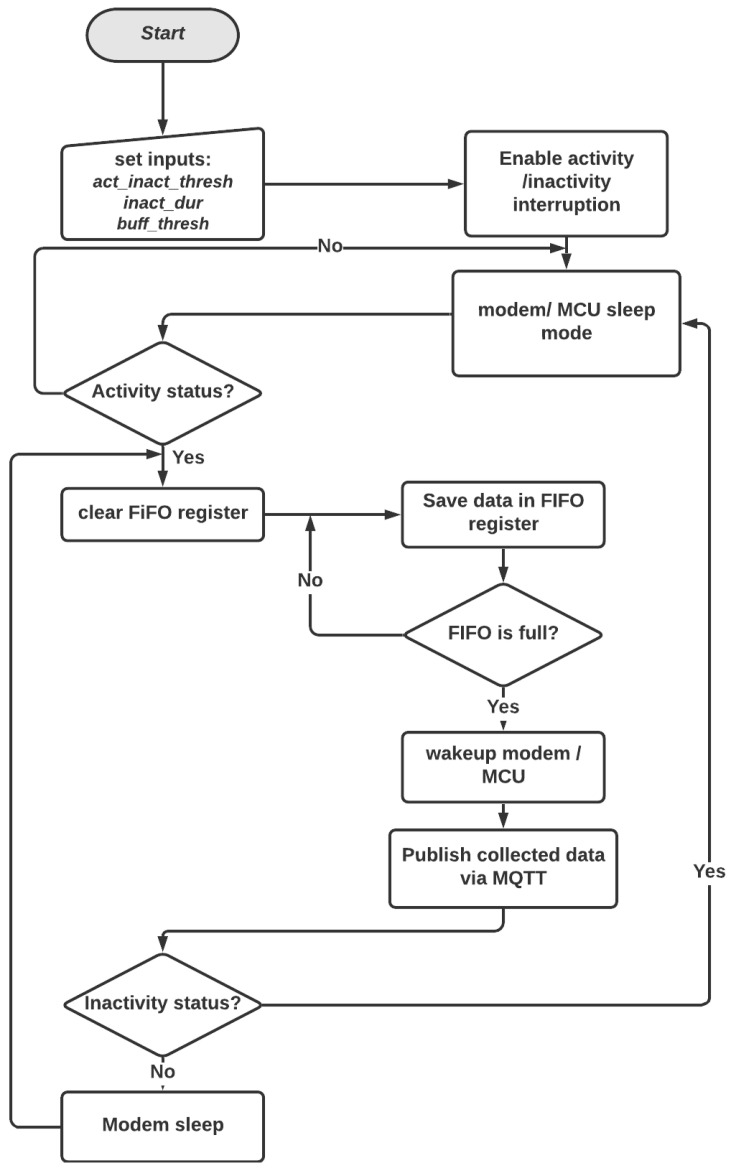
Flowchart of the power management block.

The Power Management Block (PMB) component represents the main block as it assures a more power-efficient system. Commonly, most commercialized accelerometer (IMU) sensors are equipped with various personalized functionality using predefined registers. In this block, we took advantage of the sensor’s interruptions possibility using specific registers to ensure a more power-aware sensor. The flowchart illustrated in Figure 2 is an overview of PMB. The activity status interruption will be triggered if the absolute value of all the accelerometer axes exceeds the predefined threshold (*act_inact_thresh*). On the other hand, the inactivity interruption will be generated if *act_inact_thresh* stays in exceeded more than a prescribed duration (*inact_dur*). According to Figure 2, all the wearable device components will be first initialized on sleep mode (low energy), namely, the used accelerometer, the Wifi module, and the Microcontroller Unit (MCU). When a movement or an activity is detected based on *act_inact_thresh*, the sensor will trigger an activity event. Therefore, the First-In, First-Out (FIFO) register will be enabled and initialized to buffer a time window of samples (each axis of the accelerometer). An interruption will be invoked when the buffer is full (*buff_thresh*) to send all the acquired samples to the MCU at a time. The FIFO register can help reduce the delay of each data transmission. Accordingly, the WiFi modem will be powered on to send the acquired data via MQTT. The operation will be repeated until an inactivity event is invoked when *inact_dur* is elapsed.

### 3.2. Message Queuing Telemetry Transport Broker (MQTT)

MQTT is a lightweight messaging protocol mainly dedicated to IoT applications. Unlike the client/server rule used with HTTP (Hypertext Transfer Protocol) protocol, MQTT uses a single publication/subscription for faster and more secure communication. Therefore, several components connect to a single MQTT server or Broker to either publish information or subscribe to it. The MQTT broker plays an essential role in our proposed solution, linking all system components and exchanging messages loosely. It allows the independence between sensors and the applications that analyze the collected data. Accordingly, if the system is in activity mode, every set of data received from the PMB will be published via MQTT broker (*Acc_data*) as well as the activity state (*Event_mode*); the analysis component (SWB (Sliding Window Block) + PRDB (Prediction Block)) subscribe to those topics and predict if a fall is identified. The system will enter stand-by mode if an inactivity state is detected.

### 3.3. Sliding Window Block (SWB)

The sliding window technique presents a fundamental process for enhancing event detection when facing time complexity. In ML models, two types of windowing are introduced: a fixed-length non-overlapping sliding window (FNSW) and a fixed-length overlapping sliding window (FOSW). In our work, we have implemented the FOSW model for SWB, as it is the most suitable for a real-time constraint by covering several time segments for better precision as proven in [31]. The received data *Acc_data* will then be combined and windowed into equal time frames using a predefined overlap rate (*Win_data*).

### 3.4. Prediction Block (PRDB)

The PRDB performs the core of the analysis component’s reliability. Hence, we have taken advantage of our previously introduced research works based on the SDL models that have demonstrated significant effectiveness in handling additive noises that used sensors may face [17]. The objective of the SDL algorithm is to frame the low-dimensional training data **X** = [$x_{1}$, …, $x_{N}$] of *N* samples to a high and sparse dimensional representation denoted **A** using a learned and optimized dictionary **D**, in order to seek a further discriminated pattern, and it is easier to be distinguished through an optimization problem generally defined by the following Equation (1):

(1) $$\begin{array}{c} \arg\;\min_{D,A}\{\sum_{i=1}^{N}(\frac{1}{2}\left|\right|x_{i}-Da_{i}{\left|\right|}_{2}^{2}+\lambda_{1}\left|\right|a_{i}{\left|\right|}_{q})+\lambda_{2}f_{A}\left(A\right)+\lambda_{3}f_{D}\left(D\right)\}, \end{array}$$

in which the function $f_{A}\left(.\right)$ could be a logistic function, a linear classifier, a label consistency term, a low-rank constraint, or a Fisher discrimination criterion. The term $f_{D}\left(.\right)$ forces the incoherence of the dictionary for different classes [32]. The $\lambda_{1}$ defines the regularization parameter that affects the number of nonzero coefficients (normally q=1), whereas $\lambda_{2}$ and $\lambda_{3}$ are two scalar parameters that balance the importance of each associated function.

To boost the system’s accuracy, we have combined a threshold-based algorithm to check post-fall stability and our SDL-based algorithm to double-check a fall for two consecutive FOSW. The prediction process is described in Algorithm 1. Based on the system’s activity state of *Event_mode*, every *Win_data* will be normalized and prepared (*Data_preparation*()) for prediction using *Sdl_pred()* function. If the SDL algorithm has detected an occurrence of a fall, a function of a post-fall stability check will be engaged (*check_stability()*) with the previous window detection (*pre_pred*) to guarantee the prediction efficiency.
**Algorithm 1** Hybrid SDL algorithm for fall detection.     **Input** *win_data*, *event_mode*, *thresh*     **Output** *Pred_alert*1:pre_pred←False2:  ▷ pre_pred is a Boolean that presents the prediction of the previous window of data3:*loop*:4:**if** (*event_mode* == “Activity mode”) **then**5:    prep_sig←Data_preparation(win_data);6:    pred←Sdl_pred(prep_sig);7:    **if** pred=True **then**8:        **if** ((pre_pred)&&(check_stability(thresh)) **then**9:                              ▷ check_stability is a function that checks post fall stability10:           Pred_alert←“Fall”11:        **else**12:           13:           pre_pred←pred14:           Pred_alert←“Nonfall”15:    **else**16:        Pred_alert←“Nonfall”  **return** Pred_alert17:**goto** *loop*.

## 4. Evaluation and Results

This section aims to evaluate the proposed system’s effectiveness regarding energy consumption and detection efficiency. We will first present the experimental setup for the hardware and software parameters as well as the deployment process in the following subsections. We follow up by evaluating the offline and online fall detection accuracy. Finally, we will simulate the overall energy consumption of the system.

### 4.1. Experimentation Setup

#### 4.1.1. Hardware

We have explored two different hardware prototypes presenting different aspects:

- Discovery IoT kit: This is a low-power IoT sensor node based on the STM32L4 MCU series. It encompasses multiple wireless connectivity protocols and sensors in which we have enabled the Iventek ISM43362-M3G-L44 Wifi module and LSM6DSL accelerometer node;
- NodeMCU: A miniature IoT firmware based on ESP8266 Wifi module, and we have added an ADXL345 accelerometer sensor.

The remainder of our experimentation is focused on the Discovery IoT kit taking into account its low power and high-performance aspects. The NodeMCU will be a benchmark to compare energy efficiency.

#### 4.1.2. Software

In this experiment, we have set up different parameters in the previously mentioned components and blocks:

- PMB: We assume that an inactivity event can be detected after five minutes (*inact_dur*) of threshold stability of 65.5% (*act_inact_thresh*). We have limited the FIFO register buffer to 200 samples for each axis (*buff_thresh*), with a 100 Hz sampling rate;
- SWB: We previously fixed a window size of 4 s, knowing that a fall can take two to three seconds. We assume that, in order to double predict the occurrence of a fall, we have set a FOSW to 75%;
- PRDB: Taking into account the obtained result in our previous work [16,17], the Sparse Representation-based classifier has proved to have a better offline performance and robustness facing noises when trained on multiple situations of the Quadros et al. [11] dataset. Thus, we will be conducting our work to compare the best SDL performance data with our proposed enhanced Algorithm 1 in real-life simulation using a raw accelerometer. In addition, we have fixed a post-fall stability check to the same threshold of the activity/inactivity events for 2 s after the fall.

#### 4.1.3. Evaluation Metrics

This study is evaluated based on three reference evaluation metrics, namely, Accuracy (AC), Sensitivity (SE), and Specificity (SP). Accordingly, AC represents the overall true detection, SE represents the ability to detect authentic falls among all detected falls, and SP represents the capacity to detect real ADL (Activities of Daily Living) in all the detected ones.

#### 4.1.4. Dataset

The SDL algorithms were trained only on the accelerometer raw data of the dataset collected in Quadros et al.’s work [11]. In fact, the acquisition was carried out based on an Arduino UNO using an ADXL345 accelerometer sensor configured on 100 Hz for sampling rate and a 4 G scale. The dataset categorizes different fall events and ADLs. The recorded fall incident covers forward to fall, backward fall, right-side fall, left-side fall, fall after rotating the waist clockwise, and fall after rotating the waist counterclockwise. The ADLs performed activities enclose walking, clapping hands, moving an object, tying shoes, and sitting on a chair.

For this protocol, twenty-two volunteers were involved, repeating each activity three times, totaling 36 signals for each testing cycle. Thus, a total number of 792 signals become available. Half of them are related to falls, and half simulate ADLs. For each fall and ADL, the signal starts with a static position (resting arms), followed by a few steps before the event simulations. The average signal duration is 9.2 s.

The acquired signals were divided into two data sets: a training set with 600 signals (approximately 75% of all data) and a testing set with 192 remaining signals. This division was performed randomly but assuring the same proportion of each movement type from the data acquisition protocol. Thus, the training set and testing set are comprised of 50 and 16 signals, respectively, for each one of the twelve different movements defined by the data acquisition protocol.

In order to test the real-life online performance, a volunteer randomly simulated some falls, fall-like, and walking actions. The simulation included random falls, jumping, clapping hands, raising and releasing hands, and walking. The volunteer starts the simulation with a walking or resting state and then performs the desired movement and afterward walks or does other ADL movements again.

#### 4.1.5. System Scenario Overview

**Figure 3:**
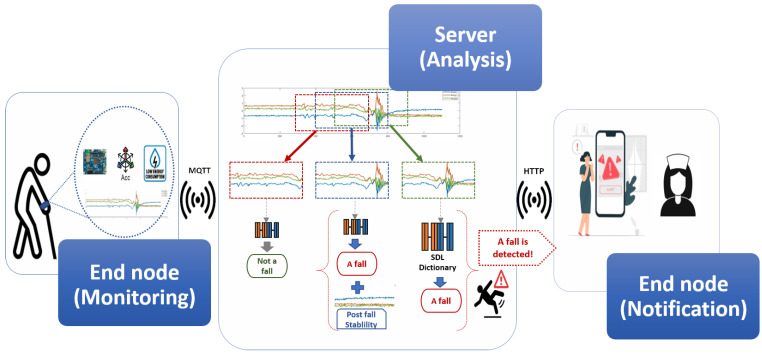
System overall scenario.

Figure 3 represents our proposed system inference explained as follows: The patient (user) must wear the device on the wrist. When the system detects an activity status, the device will be wakened up to acquire data and send it via MQTT protocol. Afterward, the data will be sliced into subsets of overlapped windows. Each window will be assessed separately by the proposed Algorithm 1. When a fall is detected for two successive windows, then an alert will be triggered to a mobile application of the supervisor or the healthcare provider.

#### 4.1.6. Deployment

**Figure 4:**
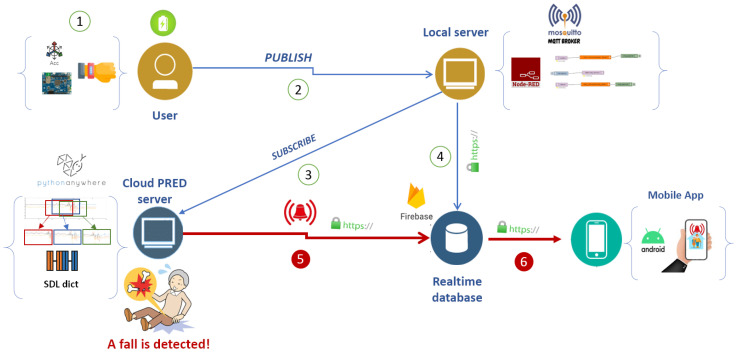
Deployment architecture of the proposed work.

Implementing our proposed MQTT-based solution prototype has followed two primary maturity processes to enhance the system’s real-time response. The proposed OWFD MQTT-based solution was deployed on a local server as the first version of a proof of concept, as illustrated in the following Figure 4. It consists of the following blocks: (i) A message broker Mosquitto for lightweight MQTT protocol, (ii) NodeRed, a programming tool to wire together hardware devices to the online services and APIs (Application Programming Interfaces), (iii) Firebase for building a real-time database and connecting APIs, and (iv) PythonAnywhere, a cloud-based environment to deploy the analyses’ components.

Second, we have suppressed the node red component to alleviate the processing of the system and connected the acquired data via MQTT directly to the Python script. Afterward, we created Docker images for the Mosquitto broker and the Python script of the analysis phase. The configuration was achieved using the Docker-Compose specification for both services. The deployment and running of the system were assured on a local server. Finally, the Firebase real-time database was used to satisfy pushing notifications into the Android mobile application.

#### 4.1.7. Mobile Application

**Figure 5:**
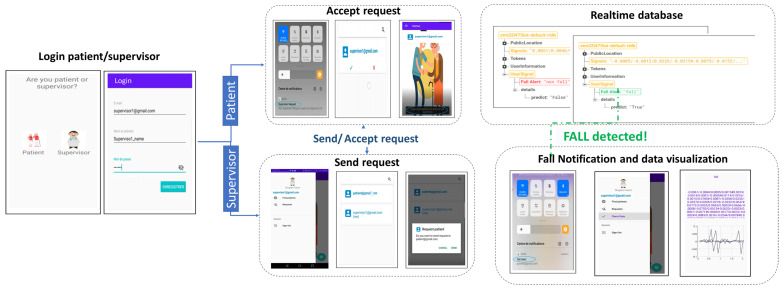
Mobile application architecture.

The mobile application illustrated in Figure 5 was developed on Android. The end user can either log in as a patient or supervisor. A supervisor must send a request to the addressed patient to enable the alert notification. This latter can either accept or decline the corresponding request. The mobile network must be enabled to maintain notification when a fall is detected. When the database acquires a fall detection alert, the fall notification will be pushed to the mobile application of the supervisors as well as the last acquired data window.

### 4.2. Evaluation of Detection Accuracy

Based on our previous works [16,17], we assume that the top three best SDL offline performances are achieved by a Sparse Representation-based Classifier (SRC) [33,34], Low-Rank Shared Dictionary (LRSDL) [35,36], and Dictionary Learning with Structured Incoherence (FDDL) [37]. This is obtained when the methods are trained on 75% of the dataset and tested on 25%. Table 1 summarizes the achieved offline result, in which SRC surpasses other SDL algorithms with an accuracy reaching 99.8%.

We have maintained the obtained Dictionary D of our offline experiments to implement it in the online experiment. In the prediction process, each received window sample will be classified to the corresponding label while maintaining a minimum residual error of the classes as shown in Equation (2):

(2) $$Label\left(x_{aq}\right)=\arg\;\min_{i}r_{i}\left(x_{aq}\right),$$

in which $x_{aq}$ is the acquired sample vector, $r_{i}=\left|\right|x_{aq}-D\sigma_{i}\left(a\right){\left|\right|}_{2}^{2}$, and $\sigma_{i}$ is the selective function of the coefficient vector associated with the class *i*.

The SDL algorithms have shown an impressive 100% sensitivity over falling impacts in different situations when tested in real-life simulations. Nevertheless, when facing a fall-like simulation such as clapping hands and jumping, the classification algorithm has shown a notable decrease in its ability to distinguish a non-fall. In favor of enhancing the real performance of the trained SDL models and reducing false alarms, we have implemented Algorithm 1 described previously. A volunteer has performed different fall-similar movements when wearing the device on the non-dominant arm multiple times. The obtained result, illustrated in Table 2, shows that, by adding the double check and post-fall phases, we have enhanced the system accuracy from 64.3% to 91%. In addition, we ameliorated the ability to distinguish real ADL and reduce false alerts from 58.3% to 89.2% of specificity while maintaining the system’s excellent 100% sensitivity performance. In conclusion, our proposed double-check hybrid SDL algorithm has shown an impressive overall enhancement, especially for unseen data when learning the SDL algorithm, i.e., going upstairs and downstairs, raising and releasing hands, as the online SDL has faced a deteriorated efficiency.

### 4.3. Energy Estimation

To demonstrate the energy efficiency of our fall detection system, we approximate the energy consumption calculation of the wearable device with [28]:

(3) $$E=V\times (I_{s}\times t_{s}+I_{r}\times t_{r}),$$

in which *E* is the total energy consumption (mJ) per second; *V* is the applied voltage supply (V); $I_{s}$ is the average consumed current while the system is in sleeping mode or idle (mA); $I_{r}$ is the average current consumption while the system is run (mA); and $t_{s}$ and $t_{r}$ correspond to the idle and operating time, respectively (s). We have compared the energy and the current in both the NodeMCU and the Discovery IoT kit. Considering the excessive power consumption of the WiFi module, we have configured it in a power-saving mode in which the WiFi modem will be off as long as there is no data ready for transmission. Table 3 shows the energy cost of each used component in both idle and operating modes. The total energy consumption is presented in the same Table 3, in which the NodeMCU components’ prototype requires almost three times more energy than the IoT kit-enabled components.

**Figure 6:**
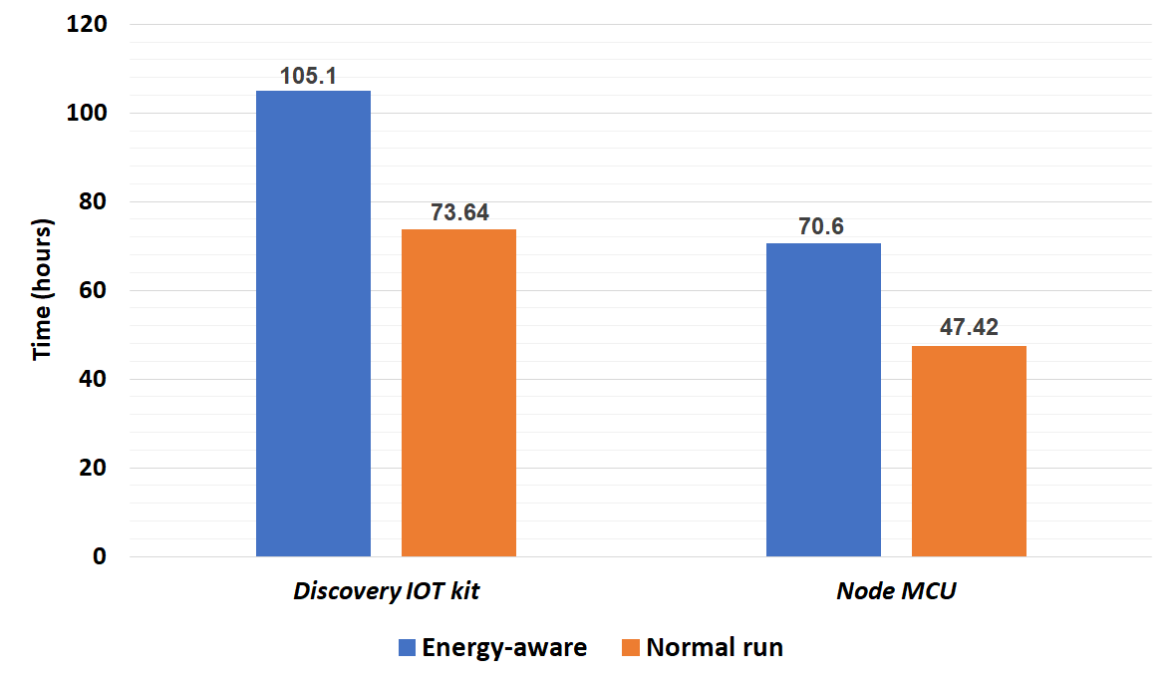
Autonomy estimation: 3800 mAH Battery.

We presume that a normal person has a minimum average of eight hours of inactivity (e.g., sleeping and resting) and 16 h of daily activity. The elderly can stagnate even more. Figure 6 demonstrates a comparison of both prototypes’ working duration when powered by a 3800 mAH battery. The proposed energy-aware system can achieve up to 31.46 and 23.18 supplementary operating hours, respectively, for the discovery IoT kit and Node MCU in both hardware prototypes used. In general, the discovery IoT kit has proven its low energy aspect compared to the node MCU reaching 105 h of autonomy for energy-aware and 73.64 h when operating normally. In addition, using the MQTT protocol with the WiFi module can save up to 10% of battery consumption per hour compared with the WiFi default HTTP protocol [38], subsequently extending the autonomy to 10 more hours when using the aware energy system with the discovery IoT kit.

### 4.4. Discussion

Designing an IoT autonomous healthcare system declares an undeniable bottleneck to fulfill strict requirements such as signal quality, energy consumption, and real-time response. In order to have a more precise overview of our proposed system, Table 4 illustrates a general comparison of the current research to the previously described related works. Table 4 shows that only our proposed IoT architecture presents a gateway-less solution via WiFi-based MQTT to assure indoor and outdoor autonomous supervision. Knowing that the WiFi module presents an excessive energy consumption, we have reached considerable battery autonomy, reaching almost five days when being active 16 h a day. The authors in [29] presented a more energy-efficient system running up to 140 h with only a 600 mAH battery using an ultra-low-power yet gateway-dependent communication protocol, i.e., Zigbee. However, the latter used more sensors (both accelerometer and gyroscope) attached to the waist. Generally, our proposed solution presents a more low-cost, easy-to-use, and fully autonomous fall detection system using an accessory-like on-body placement. Indeed, a lightweight, efficient SDL algorithm using only an accelerometer has proven a state-of-the-art accuracy for online and offline tests compared with related works.

## 5. Conclusions

Recently, fall detection solutions using an autonomous device for anywhere-anytime present significant limitations in the performance/autonomy ratio. Hence, this paper proposed a power-efficient gateway-less hybrid fall detection system. The presented IoT architecture comprises two main stages, i.e., end node and server. We focused on the wrist-based end node sensing device in which an interruption-based low-power solution was implemented to optimize data sensing and transmission. We have implemented a lightweight gateway-less MQTT communication protocol to assure high speed and good-quality data transmission to an analysis server. Subsequently, hybrid-double SDL prediction is proposed to boost system reliability, especially over fall-like movement. The system showed an overall online detection accuracy of 92.3% and can run more than 110 h with a 3800 mAH battery for a real dataset of ADLs and falls. Such a result overpasses most related works in terms of accuracy using only an accelerometer, besides showing a significantly higher autonomy than other research prototypes. Both results prove promising for real applications that require a reliable and long-term anywhere-anytime solution.

This work can be extended to a more optimized solution using different low-power methods while assuring a wide area of supervision coverage. Low Power Wide Area Networks like LoRawan present an optimal solution for a gateway-less autonomous system. Our work is built on energy consumption yet accrues a sampling rate of 100 Hz. We are anticipating a down-sampling and up-sampling solution to lower the consumption of the sensing node while keeping its efficiency. Lastly, we look forward to minimizing our preliminary prototype solution into a more low-power watch-like gadget.

## Figures and Tables

**Table 1:** Offline SDL achieved state-of-the-art performances.

SDL Algorithm	SRC	FDDL	LRSDL
AC (%)	99.8	98.9	97.4
SE (%)	100	98.9	97.9
SP (%)	99.6	98.9	96.9

**Table 2:** Online SDL vs. Hybrid SDL confusion matrix.

Simulation	SDL		Hybrid SDL	
	Fall	ADL	Fall	ADL
Falling	100	0	100	0
Jumping	60	40	20	80
Raising and releasing hand	70	30	20	80
Clapping	40	60	0	100
Walking	0	100	0	100
Going Upstairs	30	70	10	90
Going Downstairs	50	50	15	85

**Table 3:** Power consumption comparison of discovery IoT and nodeMCU solutions.

Components	Running Mode		Idle/Sleep Mode		E (mJ)
	I (mA)	V (v)	I (mA)	V (v)	
STM32L4 MCU (16 MHz)	1.6	3.6	0.56	3.6	40.063
LSM6DSL (SR: 100 Hz)	0.085	3.3	0.003	3.3	
ISM43362-M3G-L44 (WIFI)	50	3.3	4.7	3.3	
NodeMCU (ESP8266EX )	80	3.3	0.9	3.3	159.893
ADXL345 (SR: 100 Hz)	0.13	3.3	0.037	3.3	

**Table 4:** Overall state-of-the-art comparison.

Feature	[26]	[27]	[28]	[29]	[30]	Our Work
Detection Approach	RNN	LSTM	Threshold comparison	Convolution, Max-pooling and LSTM	Nonlinear SVM/Threshold	Hybrid SDL and Threshold
Used Sensors	Accelerometer	Accelerometer	Accelerometer and gyroscope	Accelerometer and gyroscope	Accelerometer	Accelerometer
Placement	Wrist	Wrist	Chest	Front waist	Thigh	Wrist
Sampling rate (Hz)	32.25	50	50	100	256	100
Gateway dependent	Yes via BLE	Yes via BLE	Yes via nRF	Yes via zigbee	Yes via BLE	No via MQTT
Energy aware	Low sampling rate (32.25 Hz)	50 Hz sampling rate	50 Hz sampling rate, SPI com bus	Interruption trigger (Free fall and FIFO)	NA	Interruption trigger (Activity/Inactivity and FIFO)
Performance (%)	Offline: AC = 99 Online: AC = 70	Offline: AC = 99.6 Online: AC = 85.2	NA	Offline: AC = 99.17, SP = 99.94 Online: AC = 98.25, SP = 87.72	Offline: SE = 98.6 SP = 99.3 Online: NA	Offline AC = 99.8, SP = 99.6 Online: AC = 91, SP = 89.2
Autonomy (Hours)/battery (mAH)	NA	NA	76/1000 Continuous run	140/600 active 8 h per day	NA	115/3800 active 16 h per day

## Data Availability

This work uses an available data, see reference [11] for data availability. More details about the data are available under Section 4.1.4.
